# Oxalate content of raw, wok‐fried, and juice made from bitter gourd fruits

**DOI:** 10.1002/fsn3.706

**Published:** 2018-10-23

**Authors:** Wen‐Chun Bong, Geoffrey Savage

**Affiliations:** ^1^ Food Group Department of Food, Wine and Molecular Biosciences Faculty of Agriculture and Life Sciences Lincoln University Canterbury New Zealand

**Keywords:** juicing, soluble and insoluble oxalates, total, wok‐frying

## Abstract

The total, soluble, and insoluble oxalate contents of fresh and wok‐fried bitter gourd (*Momordica charantia*) fruits were extracted and measured using HPLC chromatography. Frozen bitter gourds were imported from Vietnam, and two cultivars characteristic of bitter gourd fruits grown in India and Malaysia were grown locally in the North Island of New Zealand. The mean total oxalate contents of ripe fruits from Vietnam, India, and Malaysia were 85.90 ± 8.60 mg/100 g wet matter (WM), while the mean total oxalates fell to 88.06 ± 0.95 mg/100 g FM when the fruits were wok‐fried. The mean soluble oxalate content of the total oxalate was 54.42% of the ripe fruits and 58.14% of the wok‐fried fruits. The three cultivars of bitter gourds were processed into juice by the addition of standard ingredients and then processed through a screw press to remove excess fiber. The final juices had an overall mean value of 27.11 mg of total oxalates/100 g WM; the mean soluble oxalate content was 85.5% of the total, which was much higher than that measured in the cooked gourds (mean 70.7%).

## 1 INTRODUCTION

Bitter gourd or bitter melon (*Momordica charantia* L.) is an edible fruit in the *Cucurbitaceae* family. Bitter gourds have many common names: balsam pear (English), Ku gua (Mandarin), Goya (Japanese), Karella (Hindu), and Assorossie (French) (Morgan & Midmore, 2002). Bitter gourds are extensively cultivated in China, India, Japan, Vietnam, Thailand, and Malaysia. They are also cultivated in East Africa, the Caribbean, and South America (Gupta, Sharma, Gautam, & Bhadauria, 2011). The plant bears oblong or ovoid shaped fruit, 90–300 mm long, with warty looking ridges of jagged and triangular “teeth.” The edible fleshy skin ranges from light to dark green in color while the central seed cavity is filled with flat orange seeds 8–13 mm long and pith (Gupta et al., 2011). Bitter gourd fruits are available throughout the year in many Asian markets and are valued for their bitter flavor (Tan, Kha, Parks, & Roach, 2016). The bitter gourd fruit is mostly eaten at the immature stage when the fruit is green or beginning to turn yellow (Tan et al., 2016).

There is limited information on the nutritional composition of bitter gourd fruits. Yuwai, Rao, Kaluwin, Jones, and Rivetts (1991) report that bitter gourd fruits grown in Papua New Guinea fruit contain low levels of lipids (0.8 g/100 g wet matter (WM)) and contain nutritionally useful quantities of minerals and amino acids.

Cooking methods vary between regions with stir‐frying, steaming, boiling, pickling, canning, and herbal tea infusions being the most popular ways to process the fruits (Tan et al., 2016). Bitter gourd juice mixed with honey is a popular beverage in Taiwan (Tsi, Huang, Wu, & Lee, 2005). Despite the different preparation methods, cucurbitane glycosides, momordicosides K and L, and momordicines I and II contribute to the bitterness of bitter gourds and they are thought to play an important role in its health benefits (Tan et al., 2016). The bitterness of this fruit has led to the use of different processing and cooking techniques, such as soaking before cooking, to remove the bitter compounds, but it is possible that these processes will also remove bioactive compounds and anti‐nutritive compounds, such as oxalates.

Oxalate is an unwanted nutrient in human diets due to its adverse effects on health; these effects have been well documented (Massey, 2003; Noonan & Savage, 1999). Oxalate is not an essential nutrient, and it occurs in two forms, soluble, and insoluble. Soluble oxalates in foods can bind to calcium released from foods in the digestive tract; thus, reducing the absorption of calcium. The soluble oxalate that is absorbed has no metabolic function and will be excreted via the kidneys, where stones can form. Insoluble oxalates in foods are not biologically significant as they are usually eliminated in the feces (Simpson, Savage, Sherlock, & Vanhanen, 2009). Soaking and cooking food leads to the loss of soluble oxalates into the cooking water (Tan et al., 2016) resulting in fewer oxalates being absorbed or binding to calcium.

A number of food plants contain oxalates, but these tend to occur in higher concentrations in the leafy parts of vegetables rather than in the roots or stalks (Juajin, Vanhanen, Sangketkit, & Savage, 2012; Savage, Vanhanen, Mason, & Ross, 2000), and there are few reports of oxalates being found in the fleshy parts of edible fruits. Studies on the oxalate content in the leaves and fruit of bitter gourds are limited. The total and soluble oxalate contents of fresh bitter gourd leaves grown in Surin, Thailand were 1,113.3 and 171.4 mg/100 g dry matter (DM), respectively. The leaves contained remarkably high amounts of insoluble oxalate, which remained in the tissue even after boiling (Juajin et al., 2012). Ruan, Zheng, Chen, Xiao, and Peng (2013) gave a value of 35.1 ± 1.6 mg total oxalate/100 g WM for fruits grown in Guangzhou, China, while Tsi et al. (2005) measured a value of 60.21 mg total oxalate/100 g WM for mountain bitter gourds grown in Kaohsiung, Taiwan. The total, soluble, and insoluble oxalate contents of a Chinese variety of bitter gourd grown in the Nakornpathom region of Thailand had values of 71.0, 57.0, and 14.0 mg/100 g WM in the raw fruits (Judprasong, Charoenkiatkul, Sungpuag, Vasanachitt, & Nakjamanong, 2006). The soluble oxalate content of the tissue was reduced by 61.4% when the fruits were boiled. Zhou and Zhou (2014) showed that soluble oxalate of bitter gourd grown in Guangxi, China was 59.4 mg/g WM and that the soluble oxalate content in the raw fruit could be reduced by soaking in boiling water by 39.8% and by 37.4% following soaking in salt water at room temperature. In contrast, no oxalate was measured in the fruits of bitter gourds grown in Rajshahi, Bangladesh (Rahman, Anisuzzaman, Ahmed, Rafiul Islam, & Naderuzzaman, 2008). Overall, soaking may reduce the oxalate content of processed or cooked fruit while wok‐frying is unlikely to reduce the oxalate content.

Juicing has become a popular health trend in recent years. As vegetables and fruits are not heated or cooked during juicing, it is seen as an effective way to preserve the positive nutrients in the juices, but this does not allow for the possibility of reducing potentially toxic anti‐nutrients such as oxalates (Vanhanen & Savage, 2015). The preparation of vegetable juices commonly involves the addition of other fruits and vegetables to balance the taste of the final juice. This also has the effect of reducing potential toxic compounds in the final mix. Masticating juicers remove excess fiber, which has been shown to contain oxalates bound to this fraction (Vanhanen & Savage, 2015).

The objective of this study was to investigate the oxalate compositions of three popular cultivars of bitter gourds that were either grown or imported into New Zealand. In addition, the study also investigated the impact of cooking or juicing on the oxalate levels of the fruits.

## 2 MATERIALS AND METHODS

### 2.1 Source of materials and preparation

Indian‐type bitter gourd fruits grown locally in the Auckland region were purchased fresh at the Super Food Market, Central Auckland, NZ. Locally grown Malaysian‐type bitter gourd fruits were purchased fresh from The Asian Grocery, Church Corner Mall in Riccarton, Christchurch, NZ. The locally grown bitter gourd fruits were grown in greenhouses, as they are tropical plants that do not tolerate cool night air temperatures. Vietnamese‐type bitter gourd cultivars were purchased from The Asian Grocery, Church Corner, Riccarton, Christchurch. These fruits were imported from Vietnam by Marsanta Foods, Mt Wellington, Auckland. The fruits were purchased frozen at −18°C and sliced with the seeds removed.

The fresh locally grown bitter gourd fruits were sliced; the seeds removed and then chopped into 20 × 20 mm pieces. Approximately 300 g of chopped fruits was then weighed into a wok with 20 ml of canola salad oil (Sunfield, Tasti Products Ltd., Auckland, New Zealand). They were cooked on a high heat for 10 min, with constant turning, until cooked. The cooked pieces were then placed on a kitchen paper towel for two minutes to remove excess oil and moisture, allowed to cool and then placed in aluminum foil bags. The bags were then vacuum packed and stored in a deep freeze at −18°C until analysis commenced. The Vietnamese fruit were defrosted and cooked in the same way.

### 2.2 Juicing

All vegetables and fruits in addition to the bitter gourds were purchased fresh from the New World Supermarket, Lincoln, Canterbury, in April 2016. The fruits and vegetables were trimmed of nonedible parts using a stainless‐steel knife and the remaining edible portions were chopped, weighed, and processed using a masticating juicer (Oscar 9000), together with the bitter gourd fruits following the recipes shown in Table 1.

**Table 1:** Composition of the juicing mixes and recovery of juices following processing through a masticating juicer

Ingredients (g)	Bitter gourd varieties		
	Vietnamese	Indian	Malaysian
Bitter gourd	280.1	280.9	281.3
Apple	80.3	80.9	80.6
Celery	80.9	80.4	80.1
Cucumber	80.2	80.3	81.2
Lemon	50.6	51.6	50.2
Total weight of mixture	572.1	574.1	573.4
Yield of juice	336.4	378.2	414.7
Recovery of juice (%)	58.8	65.9	72.3
Dry matter of the juice (%)	5.10 ± 0.23	4.67 ± 1.04	4.98 ± 0.14

### 2.3 Dry matter

The dry matter (DM) contents of each sample of fresh and wok‐fried fruits were determined by drying in a Watvic oven to a constant weight at 105°C (AOAC, 1995; 935.10).

### 2.4 Extraction of total and soluble oxalic acids

The measurement of total and soluble oxalates followed the method outlined by Savage et al. (2000). Three replicates of 5 g of each sample of freshly chopped bitter gourd fruits and wok‐fried fruits and 5 g of the extracted juice were used to measure the total oxalate content, and three replicates were extracted to measure the soluble oxalate contents. Forty ml of 0.2 M HCl (Aristar, BDH Chemicals, Ltd., Poole, Dorset, UK) was added to flasks for the total oxalate extraction, and 40 ml of high purity water (Barnstead International, Dubuque, Iowa, USA, 18 MΩ·cm) was added for the extraction of soluble oxalates. All flasks were placed in an 80°C shaking water bath for 20 min. The solutions were then quantitatively transferred into volumetric flasks, allowed to cool and then made up to 100 ml with 0.2 M HCl and high purity water, respectively.

### 2.5 Sample analysis

The extracts in the volumetric flasks were filtered through a cellulose acetate syringe filter with a pore size of 0.45 μm (dismic‐25cs, Advantec, CA, USA) into 1 ml glass vials. The samples were analyzed with a high‐performance liquid chromatography (HPLC) system, using a 300 mm × 7.8 mm Rezex ion exclusion column (Phenomenex Inc., Torrance, CA, USA) attached to a Cation‐H guard column (Bio‐Rad, Richmond, CA, USA) held at 25°C. Analysis was performed by injecting 20 μl of sample or standard onto the column using an aqueous solution of 25 mM sulfuric acid (HPLC grade Baker Chemicals, Phillipsburg, NJ, USA) as the mobile phase, pumped isocratically at 0.6 ml/min, with peaks detected at 210 nm. The HPLC equipment consisted of a Shimadzu LC‐10AD pump, CTO‐10A column oven, SPD‐10Avp UV–vis detector (Shimadzu, Kyoto, Japan) and a Waters 717 plus auto‐sampler (Waters, Milford, MA, USA). Data acquisition and processing were undertaken using a PeakSimple Chromatography Data System (model 203) and PeakSimple software version 4.37 (SRI Instruments, Torrance, CA, USA). The oxalic acid peak was identified by comparing the retention time to a standard solution, and by spiking an already‐filtered sample containing a known amount of oxalic acid standard. The insoluble oxalate content of each sample was calculated by difference between the total and the soluble oxalate contents.

### 2.6 Standard calibration

Two standard curves of oxalic acid (99.99% oxalic acid, Sigma‐Aldrich Co., St. Louis, USA) were analyzed, using standards of the following concentrations: 1, 2, 5, 10, 15, and 25 mg/100 ml. One batch of standards was prepared in 0.2 M HCl while the other was prepared in high purity water. The acid standard curve was used for identifying and calculating the total oxalate content, while the water standard curve was used for calculating the soluble oxalate content. All blank and standard solutions were passed through a 0.45‐μm cellulose acetate filter prior to analysis.

### 2.7 Statistical analysis

All calculations were performed using Excel 2010. Genstat, Release 12.2 for Windows 7 (VSN International Ltd., Hemel Hempstead, Hertfordshire, UK) was used to determine the accumulated analysis of variance. The mean values were compared using the LSD method (*p *<* *0.05).

## 3 RESULTS

### 3.1 Raw and cooked fruit

The total, soluble, and insoluble oxalate contents, on a wet matter (WM) basis, of the raw and wok‐cooked fruits of the three cultivars of bitter gourd are shown in Table 2. The total oxalate contents of the three different cultivars of bitter gourds were significantly different (*p *<* *0.01), as also were the soluble oxalate contents (*p *<* *0.001). In both cases, the Malaysian cultivar contained higher levels of oxalates compared to the other two cultivars. Wok‐cooking the three cultivars gave a mean value of 88.06 ± 0.95 mg total oxalate/100 g WM, while the mean soluble oxalate was 66.02 ± 3.75 mg/100 g WM. The mean insoluble oxalate content of the three raw and wok‐fried cultivars was 22.04 ± 4.6 mg oxalate/100 g WM. Comparisons of the mean total and soluble oxalate contents between the raw and wok‐fried material showed that the increase in oxalate contents in the cooked samples was probably a result of the loss of moisture during the cooking process, although there was some variability between the different cultivars of bitter gourds. The mean proportion of soluble oxalates of the three cultivars in the raw bitter gourd compared to the total oxalate content was 73.72%, which was effectively unchanged after cooking (mean 74.97%).

**Table 2:** Mean oxalate contents (mg/100 g WM ± *SE*) of the raw and wok‐fried bitter gourd varieties

Parameters	Varieties (V)	Processing (P)		Significant difference			5% Least significant difference (LSD)		
		Raw	Wok‐fried	V	P	VxP	V	P	VxP
Total oxalate	Indian	83.32 ± 5.89	88.72 ± 5.66	**	NS	***	8.92	10.92	25.41
	Vietnamese	72.33 ± 2.04	89.26 ± 7.68						
	Malaysian	102.06 ± 4.42	86.19 ± 1.08						
Soluble oxalate	Indian	61.70 ± 3.84	60.39 ± 3.72	***	NS	NS	12.60	11.01	6.63
	Vietnamese	54.87 ± 4.18	64.56 ± 2.31						
	Malaysian	73.42 ± 2.44	73.12 ± 9.79						
Insoluble oxalate	Indian	21.62 ± 2.75	28.33 ± 7.05	NS	NS	**	11.73	16.36	14.17
	Vietnamese	17.46 ± 5.77	24.70 ± 6.39						
	Malaysian	28.63 ± 5.87	13.08 ± 9.73						

Notes

Values are means of triplicates ± *SE*. Significance ***p < 0.01, **p < 0.05, NS: Not significant.

### 3.2 Juices

Three replicate juice recipes for each cultivar of bitter gourd fruits were prepared (Table 1) using the same additional ingredients for each juice mixture. The raw bitter gourds made up 48.98 ± 0.04% of the total mixture of the ingredients used to make the juice. The overall mean recovery of juices using the masticating juicer was 65.6 ± 3.9%, and the remaining was discarded as a fiber fraction. The mean total oxalate content of the three juices was 27.11 ± 2.41 mg/100 g of juice (Table 3), while the mean soluble oxalate content of the three juices was 23.08 ± 1.76 mg/100 g of juices. The mean percentage soluble oxalate content of the total oxalate content of the juices made from the three cultivars of bitter gourd was 85.50 ± 3.74%. The total and soluble oxalate contents of the three juices were significantly different (*p* < 0.01) from each other, while the insoluble oxalate contents of the three juices were very low and not significantly different from each other.

**Table 3:** Mean oxalate contents (mg/100 g juice ± *SE*) of juices prepared from the three different bitter gourd varieties

Parameters	Varieties			Significant difference	LSD (5%)
	Indian	Vietnamese	Malaysian		
Total oxalate	31.53 ± 3.02	23.25 ± 1.09	26.54 ± 0.65	**	6.55
Soluble oxalate	26.59 ± 1.61	21.50 ± 0.68	21.15 ± 0.73	**	3.78
Insoluble oxalate	4.94 ± 3.81	1.75 ± 1.78	5.39 ± 1.11	NS	8.69

Values are means of triplicates ± *SE*. Significance **p < 0.05, NS: Not significant.

## 4 DISCUSSION

### 4.1 Raw and cooked fruits

The mean total oxalate contents of the raw bitter gourd fruits in this study were 85.90 ± 8.67 mg/100 g WM. This was only marginally higher than previously recorded values for this fruit, which ranged from 35.1 to 71.0 mg/100 g WM (Judprasong et al., 2006; Ruan et al., 2013; Tsi et al., 2005). The mean soluble oxalate content of the raw fruits of the three cultivars of bitter gourd analyzed in this study was 63.33 ± 5.42 mg/100 g WM. These levels in the raw fruits are not so important, as they are not eaten raw because they are bitter and not easy to chew. On a wet matter basis, the mean soluble oxalate of the three cultivars was 66.02 ± 3.75/100 g WM when the fruits were wok‐fried the usual method for cooking these fruits. It was interesting to note that wok‐frying marginally increased the total oxalate contents of wok‐fried fruit (mean raw 85.90 ± 8.67 compared to mean cooked 88.06 ± 0.95 mg/100 g WM). This small increase in total oxalate contents of the wok‐fried fruits can be explained by the loss of moisture during cooking (mean dry matter of the three raw fruits 5.53 ± 0.61% DM; cooked 16.45 ± 1.39 DM). In contrast, Judprasong et al. (2006) showed that boiling the fruit resulted in a 61.4% reduction in the soluble oxalate content of the fruit. The soluble oxalate would have been leached into the cooking water and discarded. Cooking the fruits in a wok with a small amount of oil would be a more common way to cook bitter gourd fruits in the regions where it is consumed. Overall, the oxalate contents of the wok‐fried fruits of bitter gourds were not high when compared to other commonly consumed vegetables (Noonan & Savage, 1999; Savage et al., 2000). Also, bitter gourd fruits were not usually consumed as a separate vegetable in a meal. They were often added to a dish and mixed with other components. This would suggest that current patterns of use do not pose a health risk.

### 4.2 Juice

The mean recovery of juice from the three bitter gourd cultivars was 65.67 ± 3.90%, and the mean soluble oxalate contents was 23.08 ± 1.75/100 g of juice. Overall, the juices had a mean value of 27.11 ± 1.82 mg of total oxalates/100 g WM; the mean soluble oxalate content was 85.13% of the total, which was much higher than that measured in the raw gourds (mean 54.5%). The mean content of insoluble oxalates in the juice was very low as this fraction was largely removed in the discarded fiber fraction. The consumption of a standard 200 ml glass of the extracted juice would result in the consumption of 46.1 mg/soluble oxalate. This was relatively low when compared to the consumption of 200 ml of juice prepared from spinach using the same masticating juicer, which would deliver 728.2 mg soluble oxalate/200 ml glass (Vanhanen & Savage, 2015). However, the consumption of three or four glasses of bitter gourd juice/day would lead to a consumption of between 138.3 and 184.4 mg soluble oxalate, which would be a moderate daily intake of oxalate and would become more of a risk if this level of consumption was continued over a long period of time.

## 5 CONCLUSIONS

Overall, this experiment has shown that raw and wok‐cooked bitter gourds contained moderately low levels of total and soluble oxalates. A small proportion of the oxalates in both the raw fruits and the wok‐fried fruits were insoluble oxalates. Bitter gourds can make a low‐to‐moderate oxalate‐containing juice and depending on the amounts consumed would pose little negative effect in the diet.

## CONFLICT OF INTEREST

The authors declare that they do not have any conflict of interest.

## ETHICAL REVIEW

This study does not involve any human or animal testing.
